# Editorial: Future research questions for improving COPD diagnosis and care

**DOI:** 10.3389/fmed.2024.1411350

**Published:** 2024-04-30

**Authors:** Ahmed S. Sadaka, Azmy Faisal, Justin L. Garner, Amany F. Elbehairy

**Affiliations:** ^1^Department of Chest Diseases, Faculty of Medicine, Alexandria University, Alexandria, Egypt; ^2^Department of Sport and Exercise Sciences, Manchester Metropolitan University, Manchester, United Kingdom; ^3^Royal Brompton Hospital London, London, United Kingdom; ^4^National Heart and Lung Institute, Imperial College London, London, United Kingdom

**Keywords:** COPD diagnosis, COPD therapy, COPD exacerbation, clinical physiology, sex differences

## Introduction

The prevalence and burden of chronic obstructive pulmonary disease (COPD) remain on the rise, especially with an increasingly aging population and the relative advancement in healthcare delivery systems. Over the last two decades, our knowledge and understanding of the different aspects of this heterogenous disease have evolved but key challenges in management still exist including earlier diagnosis, reducing exacerbation frequency, and identifying and managing commonly overlooked comorbidities, among others (1). This Research Topic brings together established experts and emerging future leaders to describe future research questions aiming at improving COPD diagnosis and care.

## The Research Topic articles

In the past, it was thought that COPD was a disease that mainly affects elderly men given the higher prevalence of smoking compared to women. However, as more women are currently smoking, the prevalence of COPD among women may now surpass that of men. In the current topic, Milne et al. provide a concise review discussing sex differences in COPD from biological mechanisms to therapeutic considerations. Sex differences in the lungs' and the airways' structure are the main mechanisms behind differences in symptoms, resting, and exertional functional measurements.

COPD remains underdiagnosed with symptoms commonly attributed to aging, deconditioning, or concomitant cardiac diseases. This can be especially problematic given the high prevalence of cardiac diseases, in addition to smoking being an important common risk factor for both conditions. Beyer et al. conducted a prospective pilot study of the prevalence of chronic respiratory disease and the relation between the clinical and lung function profiles among non-cardiac symptomatic patients. Those with abnormal lung function tests (14% of participants) reported dyspnoea that was relatively persistent over a follow-up period of 3 months. This highlights the important role of primary care in early identification and discerning of chronic respiratory disease especially with the aid of simple office spirometry. However, even with good-quality spirometric testing, early disease diagnosis could be missed considering the fact that pathological changes in COPD commonly affect the small airways, the so-called “silent zone” (2). In this regard, Liwsrisakun et al. attempted to explore the role of impulse oscillometry (IOS) and spirometry in COPD, and asthma. They showed higher sensitivity of IOS in assessing small airway dysfunction (SAD) among patients with normal FEV_1_/VC while spirometry was found more sensitive in those with reduced FEV_1_/VC. In line, integral to COPD management is the delivery of bronchodilator agents capable of deposition in the small airways. However, inhaler type can impact the efficacy of the treatment. Erdelyi et al. conducted a pilot study comparing drug delivery in two low-resistance inhaler devices utilizing numerical modeling in stable and exacerbated patients with severe COPD. Pulmonary deposition was around 10% superior with the use of the soft mist inhaler device compared with the 2 pMDI devices, and notably, no significant difference was observed in pulmonary deposition between stable vs. exacerbated states of COPD. These findings bring to attention the importance of personalizing inhaler prescriptions in COPD.

The ROME proposal has outlined a new approach to define and grade COPD exacerbations (E-COPD) with more focus on objective measures (3). In this Research Topic, a review by Coutu et al. focused on the utility of remote patient monitoring not only for exacerbation detection but also for monitoring diagnostic/treatment-related parameters and aiding delivery of care in stable COPD patients with more efficient proactive management. The prevention of exacerbations is a culprit risk reduction goal in COPD management, given that E-COPD is associated with a rapid progression of disease, decline of functional capacity, and increased risk of mortality. Multiple interventions have proved helpful in reducing E-COPD including vaccinations, inhaled pharmaco-therapies, and long-term Azithromycin (4). In a multicentre prospective study, Cuevas et al. probed into the functional and systemic effects of Azithromycin in frequent COPD exacerbators. They showed notable improvement in gas exchange at rest and with exercise. Also, baseline serum and sputum interleukin-8 elevation were found to be independently predictive of therapeutic response. It is worth noting that long-term azithromycin therapy was associated with a change in the pattern of microorganism isolates to more pathogenic bacteria, a finding that was previously described and worth balancing when considering offering such a treatment option (5). Distinguishing pneumonic and non-pneumonic E-COPD bears important treatment and prognostication implications. In the context of a quite similar presentation and limited sensitivity of the conventional chest x-ray, biomarkers can play an important role (6). In the BioInflame study, a set of plasma biomarkers was investigated for utility in identifying E-COPD and community-acquired pneumonia (CAP) (Jung et al.). Results showed that interleukin-6, neutrophil gelatinase-associated lipocalin and resistin are suitable markers for discrimination between E-COPD and CAP. These findings warrant validation in a larger cohort to deliver more evidence for implementation in clinical settings. In the same context, Amado et al. provided the first evidence of reduced levels of circulating MOTS-c and increased levels of Romo1 in patients with stable COPD; these micropeptides were associated with oxygen desaturation and reduced exercise capacity.

COPD is among the most common causes of hospital and intensive care unit (ICU) admissions, which puts a lot of burden on the patients and healthcare system alike. Cheng et al. shed light on different predictors of prolonged ICU stay among a large cohort of COPD patients. Including a comprehensive set of variables, they have created a normogram that can be applied for prognosticating and helping management guidance in the ICU setting. In this Research Topic, Ling et al. also reported a significant negative correlation between human serum albumin and in-hospital mortality in critically ill patients with COPD.

COPD management not only entails treating the obvious respiratory illness but should also encompass detailed phenotyping of patients as well as identifying and managing the associated comorbidities. Kaenmuang et al. investigated the prevalence, predictors, and progression of osteoporosis - as well as its treatment efficacy over 12 months. They showed an osteoporosis prevalence of 31.5% associated with elevated C-reactive protein levels and lower body mass index. Suffering from an exacerbation in the previous year was associated with almost two times increased odds of osteoporosis. These results shed light on an important commonly “silent” comorbidity that can be associated with a more complicated course of COPD (7). Obstructive sleep apnoea (OSA) is also a common association with COPD. Landete et al. identified overlap syndrome (i.e., OSA plus COPD) in 51% of their sample, which was associated with larger left carotid atherosclerotic plaques, putting patients at higher risk for developing cerebrovascular stroke.

## Conclusion

COPD is one of the most common respiratory diseases with significant morbidity and mortality worldwide. Earlier diagnosis will permit the introduction of more cost-effective and relevant therapeutic interventions with improved patient outcomes and less economic burden. This Research Topic describes the cutting-edge understanding of sex differences in COPD, common comorbid conditions associated with COPD, novel diagnostic and prognostic tools, and updates in treatment and preventive pharmacotherapy.

## Author contributions

AS: Writing—original draft, Writing—review & editing. AF: Writing—review & editing. JG: Writing—review & editing. AE: Supervision, Writing—original draft, Writing—review & editing.
